# Image dataset of infected date palm leaves by dubas insects

**DOI:** 10.1016/j.dib.2023.109371

**Published:** 2023-07-05

**Authors:** Abdullah Mazin AL-Mahmood, Haider Ismael Shahadi, Ali Retha Hasoon Khayeat

**Affiliations:** a Department of Electrical and Electronic Engineering, University of Kerbala, Karbala, Iraq; b College of Computer Science and IT, University of Kerbala, Karbala, Iraq

**Keywords:** Date palm, Dubas bug, Dubas honeydew, Ommatissus binotatus lybicus Bergevin, Palm diseases

## Abstract

This paper presents a valuable dataset that has been collected from different regions of Aoun district at Karbala Governorate in Iraq. The dataset includes different images of date palm leaves that have been collected over about six months, which represents the period of the dubas insect (Ommatissus binotatus lybicus Bergevin) growing. The collected images of the palm leaves are classified into four categories based on their health status and the stage of the insects present. These categories are named as healthy, infected with bugs only, infected with honeydew only, and infected by mixed insects and honeydew. The images of the leaves that are infected by the insects include different stages of an insect life cycle, ranging from the third generation of nymphs to the adult stage in the fifth stage of the nymph. Two types of drone cameras have been used in imaging. The presented images in this paper are 3000 images, with 800 images per non-bug category and 600 images for the bug category. The dataset provides valuable information for determining the severity and extent of the infestation, as well as for estimating the number of insects present in a given area.

**Specifications Table**

**Table:** 

Subject	Computer Science, Agricultural Sciences

Specific subject area	Computer Vision, Pattern Recognition, Segmentation, machine learning, pre-trained network, deep learning
Type of data	Images(.jpg)
How the data were acquired	Images of palm fronds have been captured with two types of digital cameras with the following specification: First type: Canon 77D 24MP CMOS Sensor, DSLR Camera, Image processor: DIGIC 7, AF System / Points:45 cross-type AF points, Type LCD Monitor: Vari angle touchscreen 3.0" Lens: 55 mm f2.8 Image size: (RAW) 6000 × 4000 Second type: Camera: Mavic Air 2 is equipped with a 48-megapixel. It also has a large 1/2-inch CMOS sensor, which makes it capable of capturing high-quality photos and videos. Image size: (RAW) 8000 × 6000
Data format	JPG
Description of data collection	The data have been collected from infected and uninfected palm fronds at Aoun area-Karbala, Iraq, during two generations of the Dubas insect. The study targeted the third, fourth, and fifth nymphal stages of the insect. High resolution images (6000 × 4000 × 3) by Canon 77D camera with a total number is 2200 images and (8000 × 6000 × 3) by DJI Mavic camera with a total number is 800 images. The images have been captured and then cropped to a size of (896 × 869 × 3). The resulting images are classified into four categories: healthy, bugs, honey, and mixed-insect/honey. The total number of resulting images is 3000, with 800 images per category for the non-bug categories and 600 images for the bug category.
Data source location	Country: Iraq, Governorate: Karbala, Cite: AL-Hussaniea, Aoun, Abo-Asead. Within this range of coordinates 32° 40′ 28.3'' N 44° 04′ 47.7'' E 32° 37′ 25.7'' N 44° 10′ 27.5'' E
Data accessibility	Repository name: Mendeley Data identification number: Direct URL to data: https://data.mendeley.com/datasets/2nh364p2bc
Related research article	Published in: 2022 International Conference on Electrical, Computer and Energy Technologies (ICECET) DOI: 10.1109/ICECET55527.2022.9872955 https://ieeexplore.ieee.org/document/9872955

## Value of the Data

- This dataset provides images of healthy and infected date palm leaves by the Dubas pest. Where the Dubas is an insect that belongs to the phylum Homoptera. Its mouth parts are piercing, absorbent, and harmful. The scientific name of the Dubas is "Ommatissus binotatus lybicus Bergevin”. It is very harmful to date palms because it absorbs the plant juices from wicker, leaves, stalks, and fruits, as this causes the fading and yellowing of these plant parts [1].
- The data can help determine the overall severity and level of date palm infection with dubas.
- This can help in calculating the expected losses in the crops [2].
- Early identification of palm infection with dubas helps to take the necessary measures for prevention and control. It also contributes to preserving palm trees and the citrus trees planted under them from diminishing, which is reflected positively on the climate [3].
- Traditional methods of diagnosis the dubas pest requires expert knowledge and spend a long time to diagnose vast cultivated areas, so this dataset can be used in automatic diagnosis of the pest
- Datasets can be used in machine learning, and deep learning to build a powerful insect taxonomy [4].
- Standalone systems for dubas pest detection and treatment can employ datasets in the training phase then use a drone for real-time diagnosis and treatments [5]. This leads to better accuracy, less effort, and reduce pesticides that positively reflect the environmental impact and cost savings [6].

## 1 Objective

The dataset is generated to address the challenge of identifying infected palm trees by Dubas insects. The goal is to create an automatic diagnosis system to reduce the time, cost, and effort involved in identifying affected palms and minimize the amount of drugs consumed during treatment.

## 2 Data Description

**Fig. 1:**
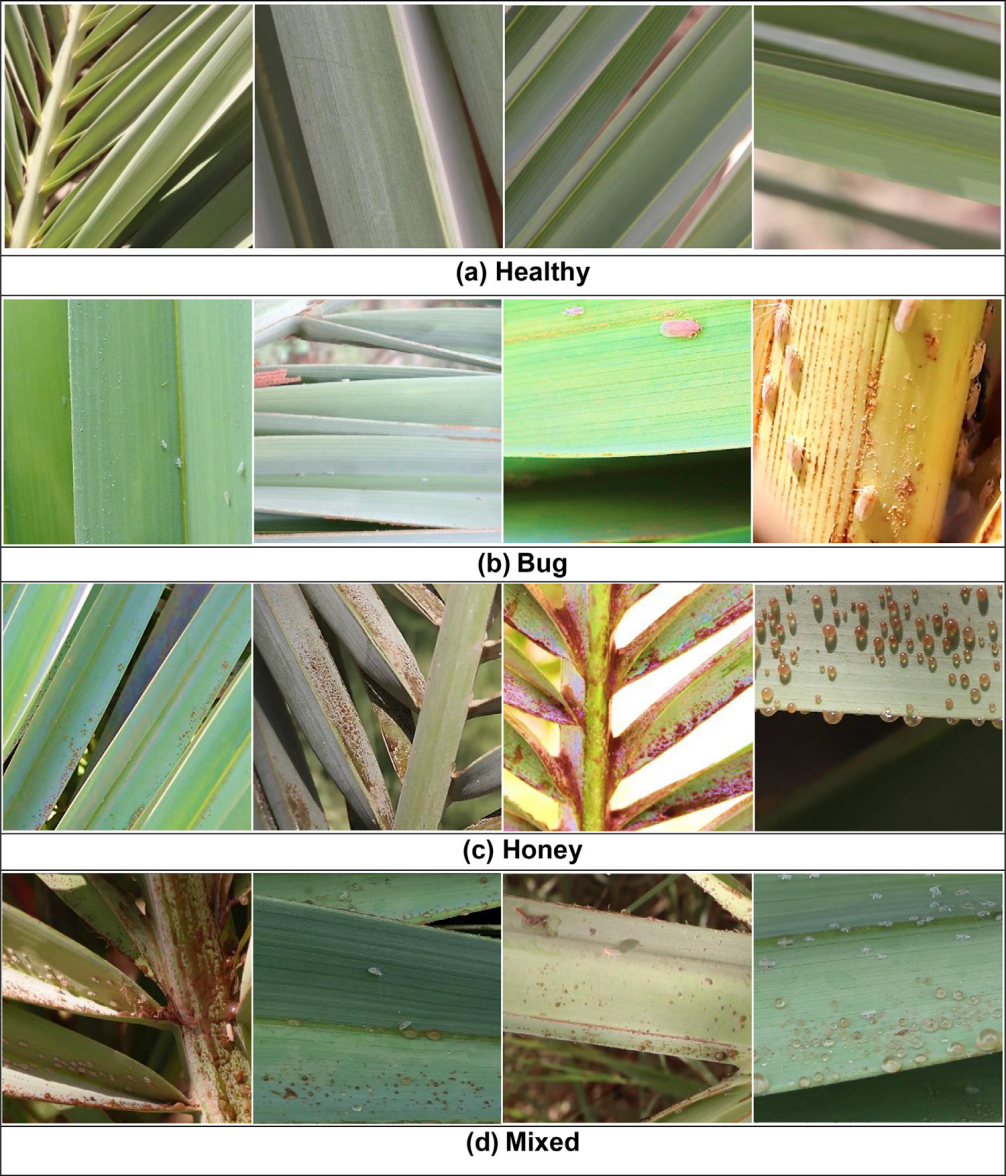
Samples of each category of date palm leaves as follows: (a) Healthy palm leaves, (b) infected leaves by dubas bug, (c) Infected leaves by honeydew, (d) Mixed - Infected leaves by both dubas bug and honeydew.

The dataset has been captured over about 6 months from different regions of Aoun district at Karbala Governorate in Iraq within ranges of coordinates (32° 40′ 28.3'' to 32° 37′ 25.7'' N and 44° 04′ 47.7'' to 44° 10′ 27.5'' E). It includes four categories of date palm leaves Images include: healthy, dubas bug, honeydew, and dubas bug with honeydew (Mixed), each folder contains 800 images, while the dubas bug folder has 600 images. The resolution of the captured images are 6000 × 4000 × 3 using a Canon 77D camera and 8000 × 6000 × 3 pixels using DJI Camera. The total size of the dataset is 23.67 GB. Due to the large file size, the most important region of the captured images are cropped into 896 × 896 × 3 pixels by a Windows cutting tool. The total size of the dataset is reduced to 938 MB. Fig. 1 shows a sample of the dataset categories.

## 3 Experimental Design, Materials and Methods

**Fig. 2:**
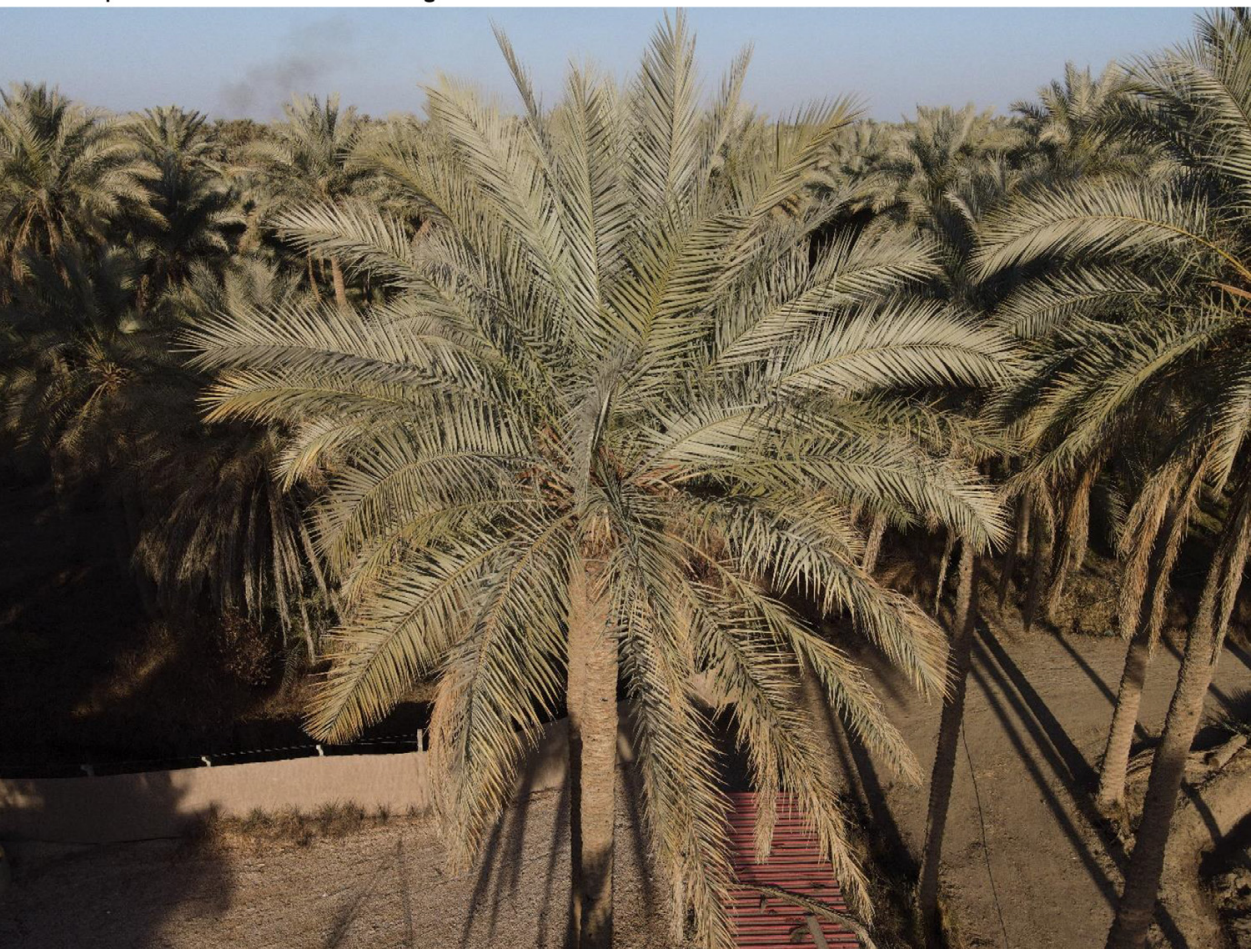
Sample of the palm orchards that are used to collect the dataset.

**Fig. 3:**
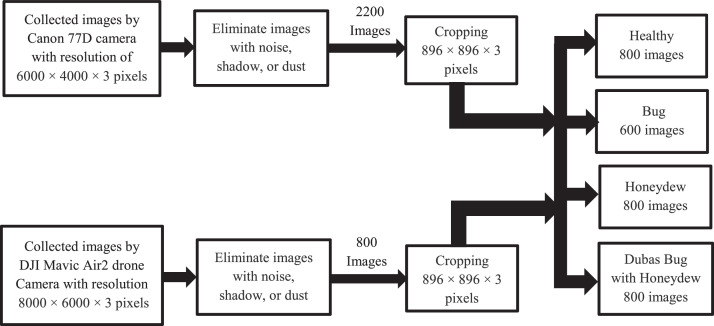
The experimental design steps.

The help of an agricultural guide has been considered in the Image acquisition of the infected palm leaves. The date palm or as named scientifically Phoenix dactylifera grows within orchards. In general, the palm orchards are jammed because of the big size (covering about 20-40 m^2^) and height (4-20 m for fruitful palms) that forms a challenge for close imaging of the palm leaves. Fig. 2 shows a sample of the palm orchards that we were imaging inside it. The data was collected during spring and autumn depending on the insect life cycle. The infected palms are detected and the drone captured images from a distance of 1-2 meters from the leaves of the palm. Other images that are captured by Canon 77D camera are captured more closely about 0.5 – 1 meter. The captured images are processed by cropping to specify the infected regions of the leaves that resulted in the final dataset images with a size of 896 × 896 × 3 pixels. According to the growth of the insect during a different season, the dataset has been divided into 4 groups (folders). In the autumn season, small bugs with eggs exist on the infected leaves. In the spring season, clear bugs appear on the infected leaves, while at the end of spring and beginning of summer, honeydew appears on the leaves. images with noise, shadow, or dust are eliminated. Fig. 3 describes the experimental design steps. All imaging has been captured with the permission and knowledge of the orchard owners.

## Ethics Statements

the study met ethical requirements by obtaining informed consent from study participants and not causing harm to live organisms. The authors also took measures to protect the privacy of the owners and the orchards.

## CRediT Author Statement

The article's authors contributed to the research in various ways, as described in the Author Contributions section.

**Abdullah Mazin AL-Mahmood:** Image capturing, data organizing, writing, investigations.

**Haider Ismail Shahadi:** Study conceptualization, writing, methodology, investigations, and supervision.

**Ali Retha Hasoon**: Study reviewing, supervision.

## Declaration of Competing Interest

The authors declare that they have no known competing financial interests or personal relationships that could have appeared to influence the work reported in this paper.

## Data Availability

Image dataset of infected date palm leaves by dubas insects (Original data) (Mendeley Data).
